# Validity and reliability of measures to assess constructs from the inner setting domain of the consolidated framework for implementation research in a pediatric clinic network implementing HPV programs

**DOI:** 10.1186/s12913-019-4021-5

**Published:** 2019-03-29

**Authors:** Timothy J. Walker, Serena A. Rodriguez, Sally W. Vernon, Lara S. Savas, Erica L. Frost, Maria E. Fernandez

**Affiliations:** 1 Center for Health Promotion and Prevention Research, Department of Health Promotion & Behavioral Sciences, University of Texas Health Science Center at Houston School of Public Health, 7000 Fannin St., Houston, TX 77030 USA; 2 Department of Population and Data Sciences, University of Texas Southwestern Medical Center, 5323 Harry Hines Blvd., Dallas, TX 75390 USA

**Keywords:** Consolidated framework for implementation research, CFIR, Inner setting, Measurement of implementation, Implementation science, Human papilloma virus, HPV

## Abstract

**Background:**

Accurate and valid measures for implementation constructs are critical to advance research and guide implementation efforts. However, there is a continued need for valid and reliable measures for implementation research. The purpose of this study was to assess the psychometric properties of measures for the Inner Setting domain of the Consolidated Framework for Implementation Research (CFIR) in a network of pediatric clinics.

**Methods:**

This study used cross-sectional survey data collected from physicians, advanced practice providers, clinic managers, and clinical staff (*n* = 546) working in a pediatric clinic network (*n* = 51). Surveys included measures assessing Inner Setting constructs from CFIR (culture, learning climate, leadership engagement, and available resources). We used a series multilevel confirmatory factor analysis (CFA) models to assess factorial validity. We also examined measure correlations to test discriminant validity and intraclass correlation coefficients, ICC(1) and ICC(2), to assess inter-rater reliability.

**Results:**

Factor loadings were high (≥0.60) for all but one of the measurement items. Most CFA models for respective constructs demonstrated adequate or good model fit (CFI > 0.90, TLI > 0.90, RMSEA< 0.08, and SRMR< 0.08). The measures also demonstrated good discriminant validity (correlations< 0.90) aside from some evidence of overlap between leadership engagement and learning climate at the clinic level (0.91). The ICC(1) values ranged from 0.05–0.16 while the ICC(2) values ranged from 0.34–0.67.

**Conclusions:**

The measures demonstrated good validity and adequate reliability with the exception of available resources, which had some evidence of lower than desired reliability and validity at the clinic level. Our findings extend previous work by providing additional psychometric evidence to support the use of these Inner Setting measures in pediatric clinics implementing human papillomavirus programs.

## Background

Effective delivery of evidence-based interventions requires an understanding of factors that influence implementation. Accurate and valid measurement of such factors is not only critical to advance research efforts but also to guide implementation in the practice setting [1]. However, recent research suggests that most measures in implementation science are not psychometrically validated [2]. There is a lack of information about whether measures capture the constructs they are intended to assess [3, 4]. In addition, psychometric testing often lacks an approach that accounts for the multilevel nature of organization-level constructs. As a result, the field of implementation science is unable to build on existing knowledge from previous studies or to effectively test the importance of theoretical constructs proposed by existing implementation models and frameworks.

There are many models and frameworks that describe factors that may influence program implementation [5, 6]. The Consolidated Framework for Implementation Research (CFIR) provides an overarching typology of factors thought to be related to implementation by organizing constructs from 19 theories, models, and frameworks into five domains: intervention characteristics, outer setting, inner setting, characteristics of the individuals involved, and the process of implementation [7]. Domains and constructs within each domain highlight the multilevel nature of program implementation by taking into account individual-, program-, and organization-level factors that impact implementation. The inner setting domain, assessed in this study, contains site-specific organization-level factors, such as culture or implementation climate, that may influence implementation in various settings. However, few measures have been developed and adequately tested that capture constructs from the inner-setting domain. As a result, there is a lack of empirical evidence informing what inner settings constructs influence implementation across different settings.

Currently, there are well-organized efforts to improve the quality of implementation-related measures [8–10]. These efforts have helped identify measures that assess constructs included in CFIR. Many of the existing measures were developed outside the context of CFIR using other frameworks [11, 12]. Therefore, they require mapping appropriate items and constructs between frameworks to measure corresponding CFIR constructs. Further, many of these measures have not been rigorously tested using multilevel analytic approaches to account for organization level constructs.

Recently, the Cancer Prevention and Control Research Network (CPCRN) developed and rigorously tested a series of measures for the inner setting domain related to the delivery of the colorectal screening programs in Federally Qualified Health Centers (FQHCs) [13, 14]. Study results revealed evidence of valid and reliable measures for culture, leadership engagement, learning climate, and available resources. Even though results were promising, more work is needed to test these measures in other clinic settings and for the delivery of other types of programs. Therefore, the purpose of this study is to expand on the original work conducted by the CPCRN by extending the application of the Fernandez et al. [14] measure, modifying it to address inner setting constructs related to human papillomavirus (HPV) vaccination, and validating the inner setting measures in a network of pediatric clinics.

## Methods

This study used cross-sectional baseline data collected as part of a pre-post intervention within a pediatric clinic network located in the greater Houston, TX area. The parent study aimed to increase adolescent (aged 11–17 years) HPV vaccination rates in network clinics. The study assessed baseline clinic rates, provider attitudes and behaviors, and clinic systems related to HPV vaccination and later implemented evidence-based interventions to increase vaccination rates. Data from physicians, advanced practice providers, clinic managers, and clinical staff were collected from July 2015 to January 2016. The University of Texas Health Science Center at Houston Institutional Review Board approved the study (HSC-SPH-15-0202).

### Recruitment and survey administration

Clinic managers, clinical staff (e.g., registered nurses, medical assistants), physicians, and other advanced practice providers (e.g., nurse practitioners, physician’s assistants) were eligible to participate in an online survey. The survey assessed attitudes and behaviors related to HPV vaccination as well as clinic-level constructs that could be associated with implementing programs to improve HPV vaccination rates. Study staff sent eligible participants an invitation and link to complete the online survey. To recruit physicians, the clinic network Chief Medical Officer announced the survey in his monthly newsletter to network physicians and followed up with an email encouraging all physicians to participate. The clinic network Chief Medical Officer also sent an email to clinic managers to introduce the survey and encourage managers and their clinic staff to complete the survey. Due to high turnover among clinical staff, study staff contacted clinic managers the week before launching the survey to verify the survey distribution list and prompt staff to check their emails for the survey. To further ensure that all clinical staff had an opportunity to complete the survey, managers were provided with survey flyers to hang in the common areas of their clinic. Physicians, advanced practice providers, clinical staff, and managers were given 1 month to complete the survey, and up to four reminders were sent to non-respondents during that time period. All data collection was anonymous, and clinic managers did not know whether staff or providers chose to complete the survey. Completion of the survey indicated consent to participate. Physicians received $50 gift cards for participation and clinic managers, clinical staff, and advanced practice providers received $40 gift cards.

### Measures

The surveys included four measures capturing constructs and sub-constructs from the CFIR inner setting domain: culture, learning climate, leadership engagement, and available resources (Table 1). These measures were originally developed (and published elsewhere) by the CPCRN to assess potential targets of implementation interventions [14]. The CPCRN selected inner setting constructs that were thought to be relevant to the clinic setting and modifiable through implementation strategies. The measures were developed in 78 FQHCs across seven states (California, Colorado, Georgia, Missouri, South Carolina, Texas, and Washington).

**Table 1:** Inner Setting construct definitions

Construct Name	Definition	# of Items
Culture	Norms, values, and basic assumptions of a given organization	7
Learning Climate	A climate in which: a) leaders express their own fallibility and need for team members’ assistance and input; b) team members feel that they are essential, valued, and knowledgeable partners in the change process; c) individuals feel psychologically safe to try new methods; and d) there is sufficient time and space for reflective thinking and evaluation (in general, not just in a single implementation)	5
Leadership Engagement	Commitment, involvement, and accountability of leaders and managers	4
Available Resources	The level of resources dedicated for implementation and on-going operations including money, training, education, physical space, and time	3

The original measures were created within the context of implementing evidence-based approaches for colorectal cancer screening in FQHCs. As a result, four of the seven original, available resources questions referred to a specific evidence-based approach to improve colorectal cancer screening. These intervention specific questions were not included as part of the available resources construct in this study. In addition, there were nine questions used to assess culture in the FQHC survey. We included seven of the original nine culture questions for this study to reduce the length of the survey. We made no other changes from the original set of measures. All questions from the inner setting constructs used a 5-point response scale ranging from 1-strongly disagree to 5-strongly agree. The following are potential score ranges for each respective construct: culture, 0–35; learning climate, 0–25; leadership engagement, 0–20; and available resources, 0–15.

### Data analysis

**Fig. 1:**
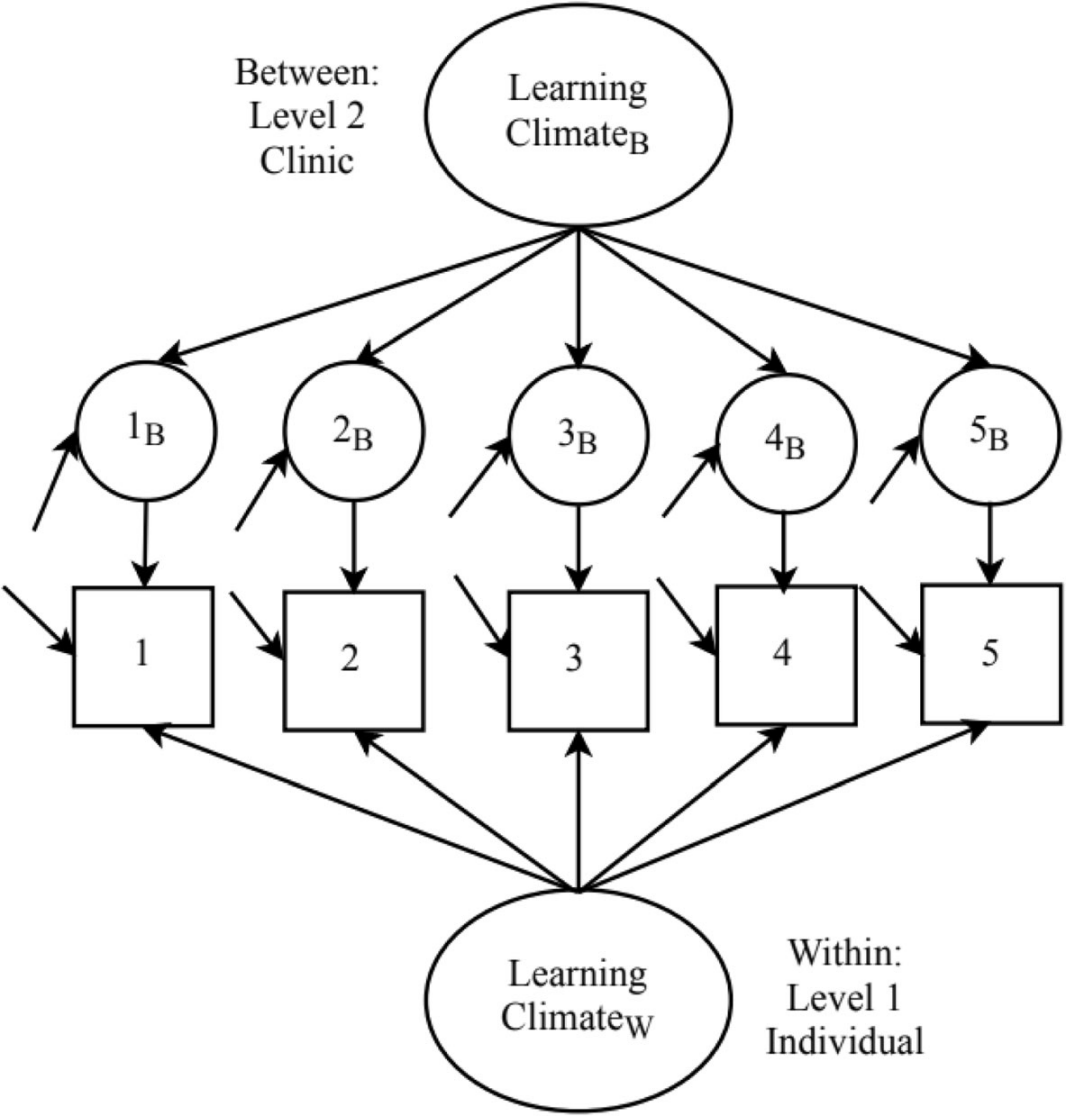
Example of multilevel confirmatory factor model for the Learning Climate Scale. The item number with B represents clinic-level items

We assessed descriptive statistics for both individuals completing the survey and clinics represented by individual respondents. We also assessed descriptive statistics for each item from inner setting constructs including item means, standard deviations, and intraclass correlation coefficients (ICC). Because we were measuring clinic-level constructs from a sample of individuals nested within clinics, we used a multilevel confirmatory factor analysis (CFA) approach to assess factorial validity [15, 16]. Using a multilevel CFA approach allowed us to model the individual and clinic level constructs simultaneously (Fig. 1).

The sample size recommendations for multilevel CFA models suggest a minimum of 50 level 2 units (or clinics). Thus, we first conducted multilevel CFA models for each measure independently. This was to keep the number of parameters estimated by each model low because there were only 51 clinics in the network. Next, we tested select construct pairs together where two factors were modeled at both the individual and clinic-levels. We chose this approach for two reasons. First, available resources had only three items so testing this construct independently would lead to a saturated model. Second, we wanted to assess some factors together to better examine relations between constructs that could have potential overlap.

Given this approach, we tested three constructs independently (culture, leadership engagement, and learning climate) and two additional models with construct pairs: 1) available resources with leadership engagement and 2) leadership engagement with learning climate. We chose these specific pairs because leadership engagement and available resources are both sub-constructs of implementation climate within the inner setting domain [7], and there is evidence that leadership engagement and learning climate are highly related constructs [14]. Because the culture measure included seven items, we tested this construct independently to avoid models where the number of parameters estimated would exceed the number of clinics, which could produce unreliable results [17].

We tested multilevel CFA models that were unrestricted where we allowed the factor loadings to freely estimate at both the individual and clinic levels. We also wanted to determine whether the factor structures were similar between the individual and clinic portions of the model. Thus, we tested a series of models with constrained factor loadings where corresponding loadings were set to be equal across individual and clinic levels. We used the Satorra-Bentler scaled chi square difference test to compare fit between constrained and unconstrained models [18].

To assess model fit, we used the collective information from the following indices: Chi square (non-significant value = good fit), comparative fit index (CFI, > 0.90 = adequate fit and > 0.95 = good fit), Tucker–Lewis index (TLI, > 0.90 = adequate fit and > 0.95 = good fit), root mean square error of approximation (RMSEA, < 0.08 = adequate fit and < 0.05 = good fit), and standardized root mean square residual (SRMR, < 0.08 = adequate fit and < 0.05 = good fit). The SRMR provides fit information about the individual (SRMR(w)) and clinic levels (SRMR(b)) for multilevel CFA models [19, 20]. We used full information maximum likelihood estimation with robust standard errors to account for non-normality of measure items and missing data. We considered model adjustments based on modification indices if they revealed substantial model improvements. All multilevel CFA models were tested using Mplus version 7.31.

We evaluated discriminant validity by calculating correlation coefficients of each pair of measures using both individual-level and clinic-level data (aggregated by clinic). We also examined correlations between factors when they were modeled together. We considered there to be a high level of overlap between factors if correlations were ≥ 0.90. If correlations were high between factors (> 0.90), we tested and compared a 1-factor solution versus a 2-factor solution.

We assessed reliability or relative consistency of responses among clinic employees. We used ICC(1) and ICC(2), which were calculated using the variance components from one-way random effects ANOVA models [21, 22]. The model components were used in the following ICC equations: 1) ICC(1) = MSB-MSW/MSB+[*k*-1)*MSW, and 2) ICC(2) = MSB-MSW/MSB where MSB is the between-group mean square, MSW is the within-group mean square, and *k* is the group size. ICC(1) values provide an estimate of variance explained by group membership where larger values indicate a greater shared perception among raters within clinics. It is typical for ICC(1) values to range from 0.10–0.30 for organization level constructs [23, 24]. ICC(2) values are an indicator of reliability of the clinic level mean scores. They vary as a function of ICC(1) and group size where larger ICC(1) values and group sizes will lead to larger ICC(2) values indicating a more reliable group mean score. We considered ICC(2) values to be in the preferred range if they were ≥ 0.70 [23, 25].

## Results

### Clinic and participant characteristics

All 51 clinics agreed to participate in the study. During the 12 months prior to data collection (July 2014–July 2015), clinics reported an average of 4030 total patient visits and an average of 2521 visits with adolescent patients (aged 11–17) over the course of the year. Almost half of adolescent patients were non-Hispanic white (47% compared to 14% non-Hispanic black, 23% Hispanic, and 16% other), and most patients had private insurance (81% compared to 19% with public insurance or no insurance).

Respondents from all 51 clinics participated in the survey. We invited 226 physicians with 129 completing the baseline survey (57% response rate). We invited 50 clinic managers, and 45 completed the survey (90% response rate). Most clinical staff and advanced practice providers invited to participate completed the survey with 420 invited and 372 completing the survey (88% response rate). Overall, the average survey response rate was 77% (range, 22–100%) with an average of 11 respondents per clinic (range, 2–36). Most participants were female, and the mean age was 40 years (Table 2). The majority of participants were non-Hispanic white or Hispanic. Clinical staff and managers were in their respective clinics for an average of 6 years.

**Table 2:** Characteristics of Survey Respondents (*n* = 546)

Variable	No. (%)	Mean (SD)
Clinic role		
Physician	129 (23.63)	–
Clinic staff	372 (68.13)	–
Clinic manager	45 (8.24)	–
Age	–	40.12 (12.46)
Sex		
Female	501 (91.76)	–
Male	45 (8.24)	–
Ethnicity		
Non-Hispanic White	187 (34.25)	–
Non-Hispanic Black	52 (9.52)	–
Hispanic	197 (36.08)	–
Native American or Alaskan Native	2 (0.37)	–
Asian	29 (5.31)	–
Native Hawaiian or Other Pacific Islander	4 (0.73)	–
Other	24 (4.40)	–
Prefer not to answer	51 (9.34)	–
Clinic staff and managers’ years in clinic		6.46 (6.99)

### Factorial validity

All inner setting constructs had complete data (*n* = 546) except for leadership engagement, which was missing responses from one respondent. All items were negatively skewed with a leptokurtic distribution. Item means ranged from 2.67 (±1.07) to 4.08 (±0.81) (Table 3). Notably, the available resources items had the lowest ICC values (0.04, 0.06, 0.04) suggesting less variance explained by the clinic level for these items compared to items measuring other constructs. However, the overall ICC values ranged from 0.04–0.16 where 15 of 19 items had values greater than 0.05, supporting the use of multilevel models [20, 26].

**Table 3:** Means (standard deviations), Intra Class Correlation Coefficients, and Standardized Factor Loadings (standard error) for Level 1 (Individual, *n* = 546) and Level 2 (Clinic, *n* = 51)

Item		N	M (SD)	ICC	L1	L2
*Culture*						
1	After trying something new, we take time to think about how it worked	546	3.71 (0.93)	0.07	0.734 (0.029)	0.959 (0.075)
2	We regularly take time to reflect on how we do things	546	3.64 (0.97)	0.09	0.600 (0.046)	0.994 (0.087)
3	Difficult problems are solved through face-to-face discussions	546	3.80 (0.89)	0.10	0.703 (0.039)	0.997 (0.181)
4	People at all levels openly talk about what is and isn’t working	546	3.81 (0.92)	0.08	0.659 (0.041)	0.992 (0.134)
5	Most people in this clinic are willing to change how they do things in response to feedback from others	546	3.80 (0.87)	0.10	0.661 (0.035)	0.995 (0.092)
6	It is hard to get things to change in our clinic^a^	546	2.67 (1.07)	0.05	0.178 (0.061)	0.954 (0.241)
7	People in this clinic operate as a real team	546	3.88 (0.89)	0.11	0.700 (0.028)	0.947 (0.095)
*Learning Climate*						
1	We regularly take time to consider ways to improve how we do things	546	3.75 (0.96)	0.05	0.644 (0.038)	0.966 (0.115)
2	People in our clinic actively seek new ways to improve how we do things	546	3.78 (0.93)	0.07	0.664 (0.043)	0.932 (0.077)
3	This clinic encourages everyone to share ideas	546	4.02 (0.80)	0.11	0.806 (0.029)	0.975 (0.035)
4	This clinic learns from its mistakes	546	4.07 (0.75)	0.09	0.823 (0.032)	0.986 (0.049)
5	When we experience a problem in the clinic, we make a serious effort to figure out what’s really going on	546	4.08 (0.81)	0.10	0.881 (0.019)	0.996 (0.039)
*Leadership Engagement*						
1	Leadership strongly supports clinic change efforts	545	3.93 (0.90)	0.14	0.909 (0.016)	0.995 (0.016)
2	Clinic leadership promotes an environment that is an enjoyable place to work	545	3.99 (0.89)	0.16	0.896 (0.022)	0.998 (0.015)
3	Leadership in this clinic creates an environment where things can be accomplished	545	3.91 (0.93)	0.16	0.955 (0.010)	0.998 (0.009)
4	The clinic leadership makes sure that we have the time and space necessary to discuss changes to improve care	545	3.88 (0.95)	0.13	0.906 (0.015)	0.989 (0.021)
*Available Resources* ^*b*^						
In general, when there is agreement that change needs to happen in the clinic, we have the necessary support in terms of:						
1	Budget or financial resources	546	3.60 (0.98)	0.04	0.804 (0.024)	0.992 (1.08)
2	Training	546	3.80 (0.93)	0.06	0.843 (0.034)	0.998 (0.315)
3	Staff	546	3.55 (1.04)	0.04	0.840 (0.026)	0.958 (0.671)

^a^Reverse Scored; ^b^estimates are based on a model that included leadership engagement as an additional latent variable

Factor loadings were high for all items (≥0.60) with the exception of question six in the culture measure where it was 0.18 at the individual level (Table 3). Loadings were consistently higher at the clinic level when compared to the individual level. This is common because the clinic level items are aggregated across respondents, which tends to lead to more reliable responses compared to the individual level [27]. All models had statistically significant chi square values demonstrating some evidence of misfit (Table 4). Fit indices from unconstrained models revealed learning climate had adequate or good fit while leadership engagement demonstrated good fit with the exception of the RMSEA value exceeding 0.08. Culture demonstrated the weakest evidence of model fit where TLI was below and RMSEA and SRMR(b) were above values indicating good fit. The unconstrained model that included available resources and leadership engagement together revealed good model fit except for the SRMR(b) value exceeding 0.08, suggesting poor fit for the clinic portion of the model. Results for the unconstrained model with leadership engagement and learning climate suggested good model fit across all indices (Table 4).

**Table 4:** Multilevel confirmatory factor analysis model fit results for constructs from the Inner Setting

Model	χ^2^	df	*p*-value	RMSEA	CFI	TLI	SRMR (w)	SRMR (b)
Culture Overall	148.88	28	< 0.001	0.089	0.917	0.876	0.046	0.087
Culture Overall Constrained	153.94	34	< 0.001	0.080	0.918	0.898	0.046	0.071
Learning Climate^b^	32.32	9	< 0.001	0.069	0.981	0.957	0.022	0.043
Learning Climate Constrained^b^	28.11	13	0.009	0.046	0.988	0.981	0.021	0.042
Leadership Engagement	23.50	4	< 0.001	0.095	0.986	0.959	0.010	0.005
Leadership Engagement Constrained	27.31	7	< 0.001	0.073	0.986	0.975	0.010	0.005
Available Resources and Leadership Engagement	79.40	42	< 0.001	0.061	0.981	0.970	0.016	0.140
Available Resources and Leadership Engagement Constrained	89.16	31	< 0.001	0.059	0.980	0.972	0.017	0.096
Learning Climate^b^ and Leadership Engagement	79.06	52	0.009	0.031	0.992	0.989	0.019	0.029
Learning Climate^b^ and Leadership Engagement Constrained	83.82	59	0.018	0.028	0.993	0.991	0.019	0.029

^b^Correlated residual variance between learning climate 1&2

Adding model constraints by setting corresponding factor loadings equal between the individual and clinic portions of the model did not negatively impact model fit. For all models, fit indices appeared to be slightly improved and in some cases moved fit indices into desired ranges (Table 4). In addition, results from Satorra-Bentler’s scaled chi square difference tests indicated no significant differences in model fit when allowing loadings to freely estimate versus constraining loadings. These results provide evidence that factor loadings were similar between the individual and clinic portions of the model.

### Discriminant validity

Correlations between constructs using individual level data ranged from 0.47–0.80 (Table 5) where learning climate and leadership engagement were the most correlated constructs. Using data aggregated at the clinic level, correlations followed the same pattern except the values were higher for each respective pair of constructs with values ranging from 0.56–0.91 (Table 5). Overall, correlations between constructs appear to demonstrate good discriminant validity with the exception of learning climate and leadership engagement where there may be some measurement overlap at the clinic level.

**Table 5:** Correlation coefficients for Inner Setting measures (individual level data below the diagonal, clinic level data above the diagonal)

Scale	Available Resources	Culture	Learning Climate	Leadership Engagement
Available Resources	1.00	0.64	0.56	0.61
Culture	0.57	1.00	0.81	0.74
Learning Climate	0.47	0.72	1.00	0.91
Leadership Engagement	0.47	0.57	0.80	1.00

Correlations using average score for each scale

We also assessed the correlation between available resources and leadership engagement from the multilevel CFA model with constrained factor loadings (data not shown). Results revealed the correlation between the constructs was 0.48 at the individual level and 0.89 at the clinic level. The clinic level value was higher in the multilevel CFA model compared to the value using aggregated data suggesting a higher degree of overlap. Results from the multilevel CFA model with leadership engagement and learning climate revealed the correlation between constructs was 0.86 at the individual level and 0.96 at the clinic level. Given the high clinic level correlation, we fit an additional model where there were two factors at the individual level and one factor at the clinic level comprised of both the learning climate and leadership engagement questions. Model fit (χ^2^ = 82.34, *df* = 53, *p* = 0.006, RMSEA = 0.032, CFI = 0.992, TLI = 0.989, SRMR(w) = 0.019, and SRMR(b) = 0.036) appeared to be similar between models with one or two factors specified at the clinic level. However, when comparing models using Satorra-Bentler’s scaled chi square difference test, results revealed having two factors at the clinic-level significantly improved fit (*p* < 0.001). This result provides evidence to support maintaining two factors at the clinic level and not collapsing the learning climate and leadership engagement questions into one factor.

### Interrater reliability

The ICC(1) values were statistically significant and ranged from 0.05–0.16 (Table 6). Available resources had the lowest value whereas leadership engagement had the highest value. Overall, these results indicate 5–16% of variance in scale scores occurred between clinics. The ICC(2) values ranged from 0.34–0.67 where they were all below the recommended levels (0.7 to 0.8 and higher) [23, 25], indicating a lower than desired level of reliability for group means. Available resources had the lowest ICC(2) suggesting poor inter-rater reliability for this construct.

**Table 6:** Clinic-level Inter-rater Reliability

Scale	ICC(1)	ICC(2)
Culture Overall	0.11*	0.56
Available resources	0.05*	0.34
Learning climate	0.11*	0.56
Leadership Engagement	0.16*	0.67

*p < 0.05

## Discussion

This study tested measures assessing dimensions of the inner setting domain of CFIR in a pediatric clinic setting. Our results suggest measures for learning climate, leadership engagement, and culture have adequate or good factorial validity. Further, tests between constrained and unconstrained models indicated that individual-level factor loadings were similar to the clinic-level factor loadings and thus provide further support to using aggregated individual responses to represent clinic-level constructs for these measures. However, there was evidence of poor validity and reliability for the available resources measure at the clinic-level. Overall, the measures demonstrated good discriminant validity with the exception of some evidence of overlap between leadership engagement and learning climate at the clinic level. In general, results indicate the measures can be used to assess CFIR constructs for clinics implementing HPV programs. While the measures demonstrated good validity, there is additional work that can be done to examine factors influencing reliability, in particular for the available resources measure.

Our results in pediatric clinics that serve a mostly insured patient population were largely consistent with results from the original CPCRN study conducted in FQHC clinics. More specifically, the factorial validity results from multilevel CFA models were similar between the sample of FQHC clinics and pediatric clinics [14]. Notably, the 7-item culture construct in our study had acceptable fit across indices. We did not include two of the nine original culture questions; however, exclusion of these questions did not appear to change the factorial validity of the measure. Therefore, using the 7-item version to measure culture is likely an acceptable form and may be preferable in surveys where limited space is available.

When synthesizing results for the available resources measure, we found that model fit was poor at the clinic-level. This finding was consistent with the fact that item ICCs for the available resources questions were low (with 2-items < 0.05) and the ICC(1) and ICC(2) values were low (0.05 and 0.34, respectively). Collectively, these results indicate a lack of consistency between raters within clinics suggesting a less reliable construct at the clinic-level for this network of pediatric clinics. The lack of consistency could be in part due to the different job types of respondents where physicians may view available resources differently than clinic managers and/or clinical staff and advanced practice providers. It is also possible there could be differing opinions from respondents within clinics about having the necessary support for budgets, training, and staff within a general context. Available resources would probably be better measured within the context of a specific intervention or program, similar to what was proposed in the original measure [14]. In the original measure, there were an additional four items that asked about available resources for implementation of a specific evidence-based approach.

The correlation results from multilevel CFA models revealed overlap between leadership engagement and learning climate. The high correlation between these constructs in the current study was also present in the FQHC study. We further examined the relation between these constructs by comparing multilevel CFA models that included one factor at the clinic-level consisting of both the leadership engagement and learning climate questions, versus two factors at the clinic-level. When comparing fit, having two factors at the clinic level appeared to improve model fit in comparison to just one factor at the clinic level. Thus, we recommend using the measures to capture the two different clinic-level constructs, which is consistent with how they were conceptualized.

In our study, ICC(1) values were slightly lower than the original FQHC study where constructs had values greater than 0.1 (range: 0.12–0.21). ICC(1) values are usually less than 0.3, but should be equal to or greater than 0.1 for items capturing an organization level construct [23, 24]. Lower ICC(1) values indicate individuals’ ratings within a clinic are less substitutable. Thus, a survey approach that targets more people per clinic may be necessary to produce more stable clinic mean scores for constructs with low ICC(1) values. For example, in our study, the ICC(2) values (reliability for clinic mean scores) tended to be higher compared to the original FQHC study. This is likely due to having more respondents per clinic (11 vs 5), which would contribute to more reliable clinic averages.

Overall, constructs had lower than desired ICC(2) values (< 0.70) where available resources demonstrated the weakest reliability of all constructs. Therefore, future research should focus on investigating factors that could impact reliability such as the ideal number of respondents per clinic or how people in different job types within a clinic respond to these clinic-level constructs. It is possible that physicians may have differing views of clinic culture than clinic staff or other providers, which could lower the inter-rater reliability. If true, the clinic-level measures for some constructs may need to be analyzed and interpreted from the viewpoint of employees in respective job types rather than aggregating scores across all clinic respondents.

There are study limitations that must be considered. This study included a relatively small number of clinics (*n* = 51). Fifty one clinics is at the low end of the acceptable range for the number of level 2 units in a multilevel CFA model [16]. As a result, we were unable to test comprehensive models (with more parameters) that included all the measured inner setting constructs together, which could help better determine the interrelations between constructs. There was also a varying number of respondents per clinic (2–36), and respondents were from different jobs within their clinic. Both of these factors could impact reliability where too few respondents within a clinic may not adequately represent the clinic, and different job types may influence respondents’ perceptions about the inner setting constructs. The clinics ranged in size, and the number of respondents per clinic was generally related to the clinic size (e.g., the smaller clinics had 2–3 respondents while the largest clinic had 36). Additionally, even though the response rate varied across clinics, only one clinic had a response rate < 50%.

Study strengths include building on existing work, using an advanced analytic approach, and having a strong response rate within clinics. Results from this study not only provide additional evidence of factorial validity for these inner setting measures, but they also extend findings to a pediatric clinic setting serving a mostly insured population. Thus, these measures have now been validated in two different clinic settings (FQHCs and pediatric clinics). This study also used a multilevel CFA approach, which allowed us to account for the nested nature of the data and assess the validity of measures capturing organization-level constructs. Our approach also allowed us to test whether the factor structures were similar between the individual- and clinic-level portions of the models, which is often assumed when using data from individuals to represent a high level construct. Finally, the average clinic response rate was high (77%) and only one clinic had a response rate < 50%, suggesting good participation across clinics.

## Conclusions

This study provides evidence for the factorial validity of inner setting measures capturing learning climate, leadership engagement, and culture in the pediatric clinic setting. Additionally, study results suggest a lack of validity at the clinic-level when assessing available resources in a general manner. Therefore, available resources may be better assessed by using questions that are framed within the context of the implementation of a specific intervention or program. When interpreting results along with the CPCRN’s original measure development and testing work [14], these measures appear to be valid in multiple clinic settings. However, more research is required to determine their validity in other public health and community settings. In addition, more work is required to determine whether respondents from different job types within a clinic (or organization) rate these organizational constructs in a similar manner. Overall, results from this study contribute to efforts aimed at improving measures for critical implementation constructs that can be used in research and practice settings.

## Abbreviations

CFA: Confirmatory factor analysis
CFI: Comparative fit index
CFIR: Consolidated Framework for Implementation Research
CPCRN: Cancer Prevention and Control Research Network
FQHCs: Federally Qualified Health Centers
HPV: Human papillomavirus
ICC: Intraclass correlation coefficient;
RMSEA: Root mean square error of approximation
SRMR: Standardized root mean square residual
TLI: Tucker-Lewis index
